# Promotion of hepatitis B virus infection by extracellular apolipoprotein E

**DOI:** 10.1128/jvi.00209-26

**Published:** 2026-06-10

**Authors:** Emad Elgendy, Sachin Kumar Tripathi, Guangxiang Luo

**Affiliations:** 1 Department of Microbiology and Immunology, Wake Forest University School of Medicine 12279, Winston-Salem, North Carolina, USA; Dartmouth College Geisel School of Medicine, Hanover, New Hampshire, USA

**Keywords:** hepatitis B virus (HBV), apolipoprotein E (apoE3), infection, attachment, entry, host-virus interaction

## Abstract

Chronic hepatitis B virus (HBV) infection remains a major burden to global health, affecting more than 250 million people worldwide. HBV and host interactions determine the HBV chronicity. Our previous studies have found that human apolipoprotein E (apoE) is incorporated in infectious HBV virions and plays important roles in HBV infection and morphogenesis. ApoE mediates HBV cell attachment through its interaction with cell surface receptors such as the low-density lipoprotein receptor (LDLR) family members and heparan sulfate proteoglycans (HSPGs). More importantly, both HBV-associated apoE and its expression in hepatocytes are critical for efficient HBV infection. However, the importance of extracellular apoE in HBV infection has not been examined. In the present study, we have determined the role of extracellular apoE in HBV infection *in vitro*. ApoE is an exchangeable apolipoprotein and was found to be efficiently transferred to HBV virions when mixed with an apoE-null HBV, as suggested by the results obtained from coimmunoprecipitation experiments. More significantly, extracellular apoE3 lipoproteins significantly promoted HBV infection in a dose-dependent manner when mixed with apoE-null HBV prior to infection. The cell culture supernatants obtained from apoE3-overexpressing HepG2 cells, sera derived from human apoE3-knockin mice, and a purified recombinant apoE expressed in *E. coli* were all able to significantly promote HBV infection. Additionally, extracellular apoE3 lipoproteins could dose-dependently enhance HBV attachment to HepG2^NTCP^ cells. Collectively, these findings demonstrate that extracellular apoE3 plays a key role in HBV infection by exchangeable transfer to the HBV envelope and mediation of HBV cell attachment.

**IMPORTANCE:**

Human apolipoprotein E (apoE) is implicated in the infection of different viruses as an entry-promoting factor. Our previous studies have demonstrated that apoE is enriched in the envelopes of hepatitis B virus (HBV) and hepatitis C virus (HCV)and promotes their infection and morphogenesis *in vitro*. In the present study, we have uncovered a previously unrecognized role of extracellular apoE3 in the promotion of HBV infection. Extracellular apoE3 was efficiently transferred to the HBV envelope when incubated with an apoE-deficient HBV. Strikingly, apoE-null HBV infection was proportionally enhanced by the increasing amounts of apoE3-containing mouse sera, as well as the supernatants of apoE3-overexpressing HepG2 cells. Likewise, a lipidated recombinant apoE3 significantly promoted HBV infection in a dose-dependent manner. Additionally, extracellular apoE3 promoted regular HBV infection. Moreover, extracellular apoE3 enhanced HBV cell attachment. These findings suggest that apoE3 lipoproteins with various densities in the plasma of chronic hepatitis B patients may play a key role in HBV infection and pathogenesis *in vivo*.

## INTRODUCTION

Hepatitis B virus (HBV) chronically infects 250–300 million people worldwide, posing one of the greatest burdens to global health (1). It is a common cause of liver fibrosis, cirrhosis, and hepatocellular carcinoma (HCC), resulting in about one million annual deaths (2, 3). Although HBV vaccine and antiviral therapies consisting of pegylated interferon-α and nucleoside analogs are available to effectively prevent and treat HBV infection, there is no functional cure for those chronically infected with HBV (4).

HBV is an enveloped DNA virus and the prototype of the *Hepadnaviridae* family. It contains a partially double-stranded DNA of approximately 3.2 kb, which is also referred to as the relaxed circular DNA (rcDNA) (5, 6). In the infected hepatocytes, the rcDNA is deproteinated prior to transport into the nucleus, where it is repaired by various enzymes to form a stable covalently closed circular DNA (cccDNA) (7–9). HBV cccDNA serves as the template for all viral RNA transcription by the cellular Pol II polymerase. It encodes seven different viral proteins: the precore (precursor of HBeAg), core (HBcAg), polymerase (Pol), three envelope proteins (L-, M-, and S-HBsAg), and the X protein (HBx) (5). The precore protein undergoes proteolytic cleavage at both N- and C-termini by cellular proteases to produce a secreted HBeAg and other intermediate proteins (10). Upon viral RNA transcription and protein expression, the terminally redundant pregenomic RNA (pgRNA) is encapsidated by the core protein as nucleocapsid within which it is converted to rcDNA by the viral reverse transcriptase (terminal protein). The rcDNA-containing capsids are assembled with HBsAg-incorporated envelopes to produce progeny virions or recycled back to the nucleus for the synthesis of additional HBV cccDNA (5).

It is believed that HBV enters hepatocytes through cell surface receptor-mediated endocytosis. Sodium taurocholate co-transporting polypeptide (NTCP) was identified as a key HBV receptor (11). Other cell surface molecules are also implicated in HBV cell attachment and entry, including heparan sulfate proteoglycans (HSPGs) and low-density lipoprotein receptor (LDLR) family proteins (12–16). HSPGs were previously found to play a significant role in HBV infection. In fact, HSPGs-binding sites were identified within HBsAg (17). One of the HSPG core proteins, glypican 5 (GPC5), was considered an HBV entry-promoting factor (16). Recently, we found that syndecan 2 (SDC2), another HSPG core protein, also acts as an HBV attachment receptor (18). The findings from our previous studies demonstrate that human LDLR plays a key role in HBV infection *in vitro* (14). It is well established that both HSPGs and LDLR family members are apoE-binding receptors (19, 20).

ApoE is primarily produced by hepatocytes in the liver and is secreted as lipoproteins of various densities, such as high-density lipoprotein (HDL), low-density lipoprotein (LDL), and very low-density lipoprotein (VLDL). It is an exchangeable apolipoprotein that plays critical roles in transport, metabolism, and homeostasis of lipids, cholesterol, and lipoproteins (19, 20). Additionally, apoE is implicated in the infection of many different viruses, including herpes simplex virus type 1 (HSV-1) (21), murine gammaherpesvirus 68 (MHV68) (22), human immunodeficiency virus (HIV) (23), HBV (24), hepatitis C virus (HCV) (25), and SARS-CoV-2 (26). Our earlier studies found that human apoE is enriched on the envelopes of both HBV and HCV. More importantly, virion-associated apoE and its expression in hepatocytes are important for HBV and HCV infection as well as virion morphogenesis *in vitro (24, 25*). Structurally, apoE consists of two distinct domains: the N-terminal receptor-binding domain (residues 1–191) and the C-terminal lipid-anchorage domain (residues 222–299), which are connected by a flexible linker region (27, 28). Through mutagenesis analysis, we have further shown that the receptor-binding pocket of apoE mediates HCV infection, whereas its C-terminal domain is important for HCV morphogenesis (29–31). Others have shown that recombinant apoE could promote HCV infection through extracellular binding to HCV virions (32). However, the role of extracellular apoE in HBV infection has not been experimentally examined. In the present study, we sought to determine the importance and underlying molecular mechanism of extracellular apoE in HBV infection. Interestingly, extracellular apoE efficiently transferred to HBV virions upon their incubation, as determined by coimmunoprecipitation experiments. More significantly, extracellular apoE markedly enhanced HBV infection in a dose-dependent manner. The results from cell attachment study indicate that extracellular apoE promoted HBV binding to hepatocytes. Collectively, our findings suggest that extracellular apoE may play a key role in HBV infection and pathogenesis *in vivo*.

## RESULTS

### Efficient transfer of extracellular apoE to HBV virions

**Fig 1:**
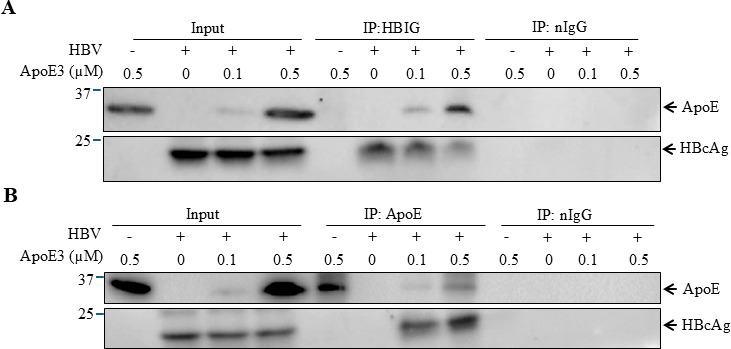
Determination of extracellular apoE3 transferred to HBV by co-IP. ApoE-null HBV was incubated with increasing concentrations (0, 0.1, and 0.5 µM) of extracellular ApoE3 present in cell culture supernatants of an apoE3-overexpressing HepG2 cell line. After 6-h incubation, HBV and apoE3 mixtures were subjected to immunoprecipitation using HBsAg-specific HBIG (**A**) or an apoE-specific monoclonal antibody (**B**). Normal mouse and human IgGs were used as controls. Immunoprecipitated complexes were loaded onto 4%–20% SDS-PAGE for electrophoresis. Proteins were transferred to the PVDF membrane, followed by immunoblotting analysis. HBcAg was detected using a HBcAg-specific antibody, whereas apoE3 was detected using an apoE-specific monoclonal antibody, WuE4. Protein marker sizes are indicated on the left (25 and 37 kDa).

Human apoE was previously found to be enriched in the HBV envelope, whose concentrations strongly correlated with HBV infectivity (24). ApoE is abundantly secreted from the liver to the bloodstream of hepatitis B patients in the forms of lipoproteins with various densities (HDL, LDL, and VLDL). It is an exchangeable apolipoprotein that rapidly and spontaneously transfers between different lipoprotein particles (33). The question arose whether extracellular apoE can efficiently transfer to HBV virions and promote HBV infection. This was examined by the incubation of an apoE-deficient HBV with extracellular apoE. The apoE-deficient HBV was produced from an apoE-knockout HepAD38 cell line as described previously (24). Human apoE3 was obtained from an apoE3-overexpressing HepG2 cell line, in which the endogenous apoE was knocked out as reported in our earlier work. The levels of apoE3 in the cell culture supernatants were quantified by EIA using a purified recombinant apoE3 as a standard. Then, apoE-deficient HBV and apoE3-containing cell culture supernatants were incubated at room temperature for 6 h. The HBV and apoE3 mixtures were subjected to coimmunoprecipitation (co-IP) using apoE- and HBsAg-specific antibodies mAb23 and HBIG, respectively. When HBsAg-specific antibody (HBIG) was used for IP, apoE3 was detected in a dose-dependent manner (Fig. 1A), suggesting that extracellular apoE3 efficiently transferred to HBV envelopes. Likewise, HBV virions were fully pulled down by an apoE monoclonal antibody (mAb23), as shown by the detection of HBcAg (Fig. 1B). In contrast, normal human or mouse IgG did not bring down any significant amount of HBV virions (Fig. 1). Taken together, these data demonstrate that extracellular apoE can efficiently transfer to HBV virions.

### Enhancement of HBV infection by extracellular apoE3

**Fig 2:**
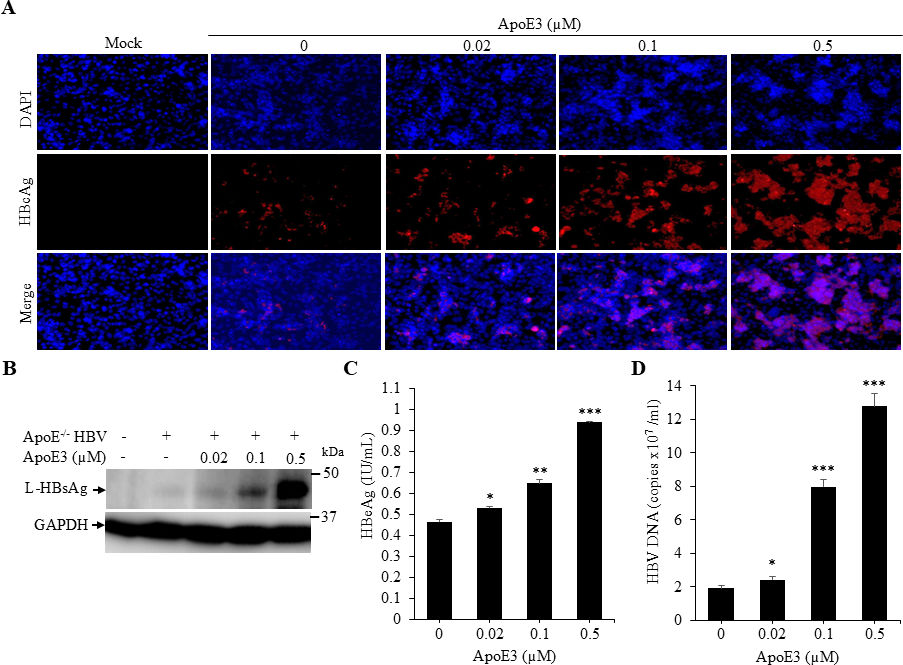
Enhancement of HBV infection by extracellular apoE3 in cell culture supernatants. ApoE-null HBV was incubated with varying concentrations (0, 0.02, 0.1, and 0.5 µM) of extracellular apoE3 present in cell culture supernatants at room temperature (RT) for 30 min. The HBV and apoE3 mixtures were used to infect HepG2^NTCP^ cells. After culturing at 37°C for 4 days post-infection (p.i.), HBcAg and L-HBsAg in the HBV-infected cells were determined by IFA (**A**) and western blot (WB) analysis (**B**), respectively. The levels of HBeAg (**C**) and HBV DNA (**D**) in the cell culture supernatants were quantified by EIA and qPCR, respectively. Data represent the mean ± standard deviation (SD) from three independent experiments. *P < 0.05, **P < 0.01, ***P < 0.001. Protein marker sizes are indicated on the right of panel B.

We have previously shown that HBV-associated apoE mediates HBV cell attachment through interactions with apoE-binding receptors, HSPGs and LDLR, on the surface of hepatocytes (14, 18, 24). It is conceivable that extracellular apoE incorporated in HBV virions would enhance HBV infection. This possibility was initially evaluated by mixing an apoE-null HBV with increasing concentrations (0, 0.02, 0.1, and 0.5 µM) of extracellular apoE3 present in cell culture supernatants of an apoE3-overexpressing HepG2 cell line prior to the infection of HepG2^NTCP^ cells. After 30-min incubation at room temperature, the HBV and apoE3 mixtures were used to infect HepG2^NTCP^ cells for 12 h. After 4 days of post-infection (p.i.), HBV-infected cells were stained for HBcAg by an immunofluorescence assay (IFA) using a HBcAg-specific monoclonal antibody (C1–5) (Fig. 2A). The levels of L-HBsAg in the HBV-infected cells were determined by a western blot (WB) analysis using a preS1-specific monoclonal antibody (Fig. 2B). The results from both IFA and WB analyses clearly show that extracellular apoE3 significantly increased HBV infection in a dose-dependent manner (Fig. 2A and B). Likewise, the levels of HBeAg and HBV DNA in the cell culture supernatants were about 2-fold and 6.7-fold higher at 0.5 μM of apoE3, respectively, compared to HBV infection in the absence of extracellular apoE3 (Fig. 2C and D). These results indicate that extracellular apoE3 transferred to the HBV virion indeed enhanced HBV infection *in vitro*.

**Fig 3:**
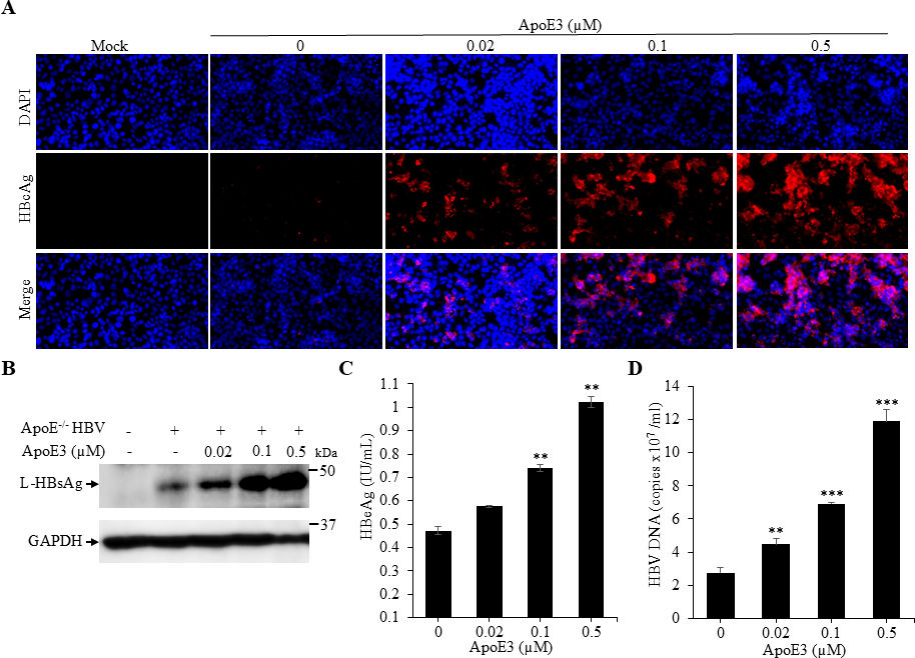
Enhancement of HBV infection by apoE3-KI mouse sera. ApoE-null HBV was incubated with varying concentrations (0, 0.02, 0.1, and 0.5 µM) of extracellular apoE3 present in apoE3-KI mouse sera at RT for 30 min. The HBV (MOI of 600) and apoE3-containing sera mixtures were used to infect HepG2^NTCP^ cells at 37°C for 12 h. After 4 days p.i., HBcAg (**A**) and L-HBsAg (**B**) in the HBV-infected HepG2^NTCP^ cells were determined by IFA and WB analyses, respectively. The levels of HBeAg and HBV DNA in the cell culture supernatants were quantified by EIA (**C**) and qPCR (**D**), respectively. Data represent mean ± SD from three independent experiments. **P < 0.01, ***P < 0.001. Protein marker sizes are indicated on the right of panel B.

To demonstrate physiological relevance closely related to humans, we determined the role of extracellular apoE3 present in the mouse serum obtained from human apoE3-knockin mice using the above-described HBV infection assay (Fig. 2). ApoE-deficient HBV (apoE^-/-^ HBV) itself was poorly infectious, given the barely detectable levels of HBcAg by IFA (Fig. 3A) and L-HBsAg by WB analysis (Fig. 3B). Strikingly, HBV infection was proportionally enhanced by increasing concentrations of extracellular apoE3 lipoproteins (Fig. 3). The apoE3 lipoproteins present in mouse sera increased the levels of L-HBsAg in infected cells by about 4-fold (Fig. 3B) and HBeAg (Fig. 3C) and HBV DNA (Fig. 3D) in the supernatants by greater than 2-fold and 4-fold, respectively, at 0.5 μM concentration of apoE3. These results are consistent with those derived from the experiments with extracellular apoE3 present in cell culture supernatants (Fig. 2), suggesting that the increased HBV infection was contributed by extracellular apoE3 transferred to HBV virions.

**Fig 4:**
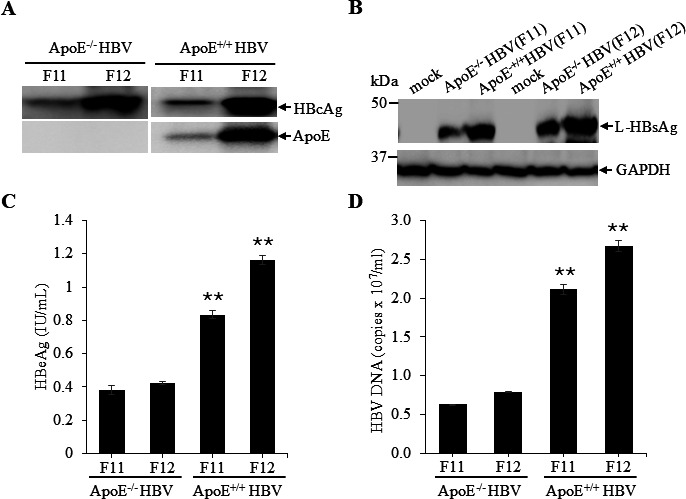
Infectivity of purified apoE-deficient and apoE-enriched HBV. ApoE-deficient HBV and its mixture with ApoE3-knockin mouse sera were purified by cesium chloride (CsCl) gradient ultracentrifugation, followed by fractionation from bottom (F16) to top (F1). Fractions 11 (F11) and 12 (F12), which contained most HBV virions, were chosen for the comparison of apoE-deficient HBV with the apoE3-transferred HBV. The levels of HBcAg and apoE in F11 and F12 were determined by WB as described in Materials and Methods (**A**). HBV with or without apoE3 transfer in F11 and F12 was used to infect HepG2^NTCP^ cells at an MOI of 100 HBV genome equivalents (100 GEq). The levels of L-HBsAg in HBV-infected cells were determined by WB (**B**). The levels of HBeAg (**C**) and HBV DNA (**D**) were quantified by EIA and qPCR, respectively. Protein marker sizes are indicated on the left of panel B. **P < 0.01.

To further validate that the enhanced HBV infectivity by extracellular apoE was attributed to virion-associated apoE instead of free apoE-containing lipoproteins, both apoE-deficient and apoE-enriched HBV virions were purified by cesium chloride gradient ultracentrifugation, as described in our earlier work (24). A total of 16 fractions were collected from bottom (F16) to top (F1). Fractions 11 (F11) and 12 (F12) contained most HBV virions, as determined by the levels of HBcAg (Fig. 4A) and HBV DNA (not shown). Likewise, apoE was detected in F11 and F12 only when the apoE-deficient HBV was mixed with the apoE3-containing mouse serum (Fig. 4A). The levels of apoE in F11 and F12 were proportional to the levels of HBcAg (Fig. 4A), suggesting that apoE in the mouse serum efficiently transferred to HBV virions. Accordingly, F11 and F12 were chosen for comparison of HBV infectivity between apoE-deficient HBV and HBV transferred with apoE from the apoE-KI mouse serum. Again, HBV with apoE transfer had much higher infectivity, as determined by about 3-fold to 4-fold higher levels of L-HBsAg (Fig. 4B) in HBV-infected cells as well as HBeAg (Fig. 4C) and HBV DNA (Fig. 4D) in the cell culture supernatants. These results suggest that apoE transferred to HBV virions contributed to enhanced HBV infectivity.

### Promotion of HBV infection by a purified recombinant apoE3 in lipidation with POPC

**Fig 5:**
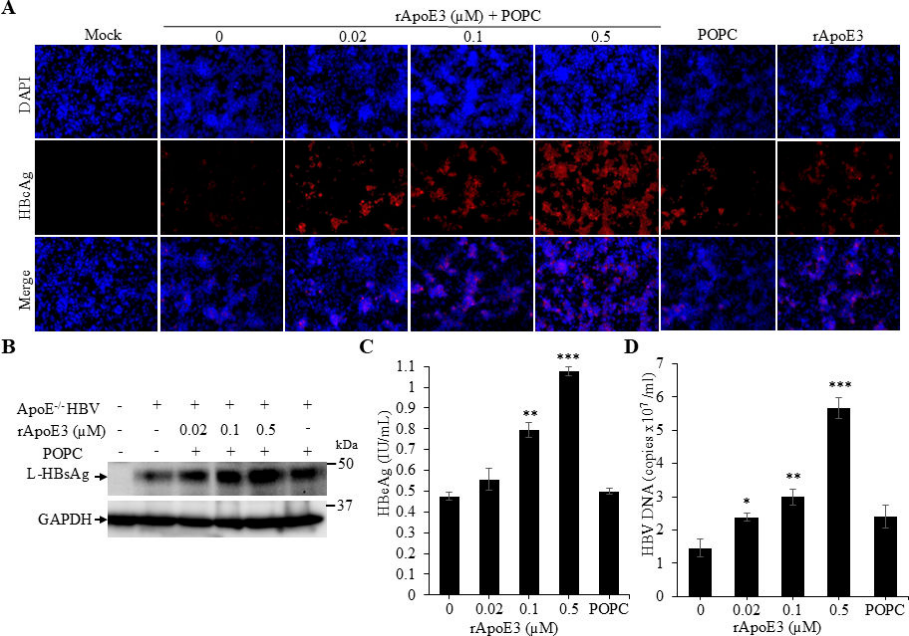
Increase in HBV infectivity by a reconstituted apoE3 lipoprotein. An apoE3 lipoprotein was reconstituted by mixing a purified recombinant apoE3 (rApoE3) with POPC at a molar ratio of 1:50, as previously described (34). ApoE-null HBV was then incubated with increasing concentrations (0.02, 0.1, and 0.5 µM) of the reconstituted apoE3 lipoprotein at RT for 30 min. POPC and rApoE3 alone were used as controls. HBV mixed with POPC, rApoE3, or both (rApoE3 + POPC) was used to infect HepG2^NTCP^ cells. After 4 days p.i., HBcAg (**A**) and L-HBsAg (**B**) expressions in the infected cells were determined by IFA and WB analyses, respectively. The levels of HBeAg (**C**) and HBV DNA (**D**) in the cell culture supernatants were quantified by EIA and qPCR, respectively. Data represent the mean ± SD from three independent experiments. *P < 0.05, **P < 0.01, ***P < 0.001. Protein marker sizes are indicated on the right of panel B.

To exclude the possible nonspecific effects associated with cell culture supernatants or mouse sera and to validate the importance of extracellular apoE3 in the promotion of HBV infection, we examined a purified recombinant apoE3 protein expressed in *E. coli* using the HBV infection assay. We initially used purified apoE3 protein alone in HBV infection, which was not fully functional to promote HBV infection (Fig. 5A). Recombinant apoE protein itself tends to form aggregates (34). Therefore, the recombinant apoE3 was reconstituted as lipoproteins by mixing it with the lipid POPC at a molar ratio of 1:50 between apoE3 and POPC according to a previous report (34). The apoE-null HBV was then incubated with increasing concentrations (0, 0.02, 0.1, and 0.5 µM) of the apoE3/POPC lipoprotein at room temperature for 30 min prior to infection. Again, the purified recombinant apoE3 in the form of lipoprotein exhibited a dose-dependent promotion of HBV infection (Fig. 5), as determined by the increasing levels of HBcAg (Fig. 5A) and L-HBsAg (Fig. 5B) in the HBV-infected cells, as well as the levels of HBeAg (Fig. 5C) and HBV DNA (Fig. 5D) in the cell culture supernatants similar to extracellular apoE3 present in cell culture supernatants (Fig. 2) and mouse sera (Fig. 3). These results demonstrate that extracellular apoE3 transferred to HBV resulted in significant enhancement of HBV infectivity in cell culture.

### Enhanced infectivity of regular HBV by extracellular apoE3

**Fig 6:**
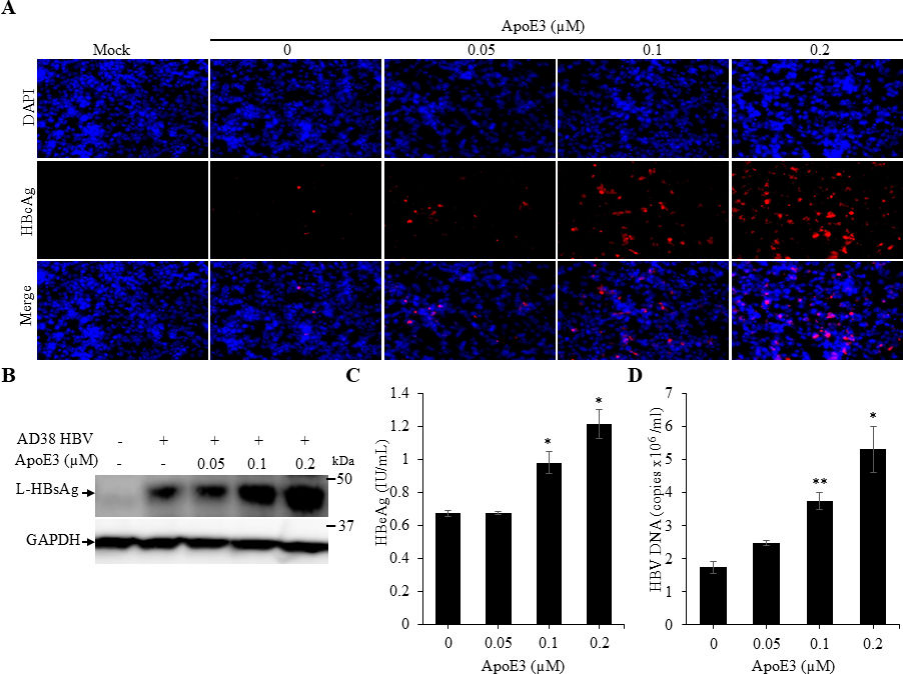
Promotion of regular HBV infectivity by extracellular apoE from apoE3-KI mouse sera. HBV produced from HepAD38 cells was incubated with varying concentrations (0, 0.05, 0.1, and 0.2 µM) of apoE3-KI mouse sera at RT for 30 min. The mixture of HBV and apoE3-KI mouse sera at an MOI of 200 was added to HepG2^NTCP^ cells with 12 h incubation at 37°C. After removal of unbound virus and washing with phosphate-buffered saline (PBS), HBV-infected HepG2^NTCP^ cells were cultured for 4 days. HBcAg expression in HBV-infected cells was detected by IFA (**A**). The levels of L-HBsAg in the cell were measured by WB analysis (**B**). The levels of HBeAg (**C**) and HBV DNA (**D**) in the cell culture supernatants were quantified by EIA and qPCR, respectively. Data represent the mean ± standard deviation from three independent experiments. *P < 0.05, **P < 0.01. Protein marker sizes are indicated on the right of panel B.

To recapitulate HBV infection *in vivo*, we sought to determine whether extracellular apoE3 could further enhance the infectivity of regular HBV that contains endogenous apoE3. HBV was produced from HepAD38 cells that express normal levels of endogenous apoE3 (24). Like apoE-deficient HBV, the infection of regular HBV was also significantly enhanced by increasing concentrations of extracellular apoE3 lipoproteins present in mouse sera, as shown by IFA staining of HBcAg (Fig. 6A). Similarly, the levels of L-HBsAg in the cell increased up to 3-fold (Fig. 6B). Likewise, the levels of HBeAg (Fig. 6C) and HBV DNA (Fig. 6D) in the cell culture supernatants increased by 2-fold to 3-fold. These findings suggest that extracellular apoE3 plays a key role in HBV infection and pathogenesis *in vivo*.

### Promotion of HBV cell attachment by extracellular apoE3

**Fig 7:**
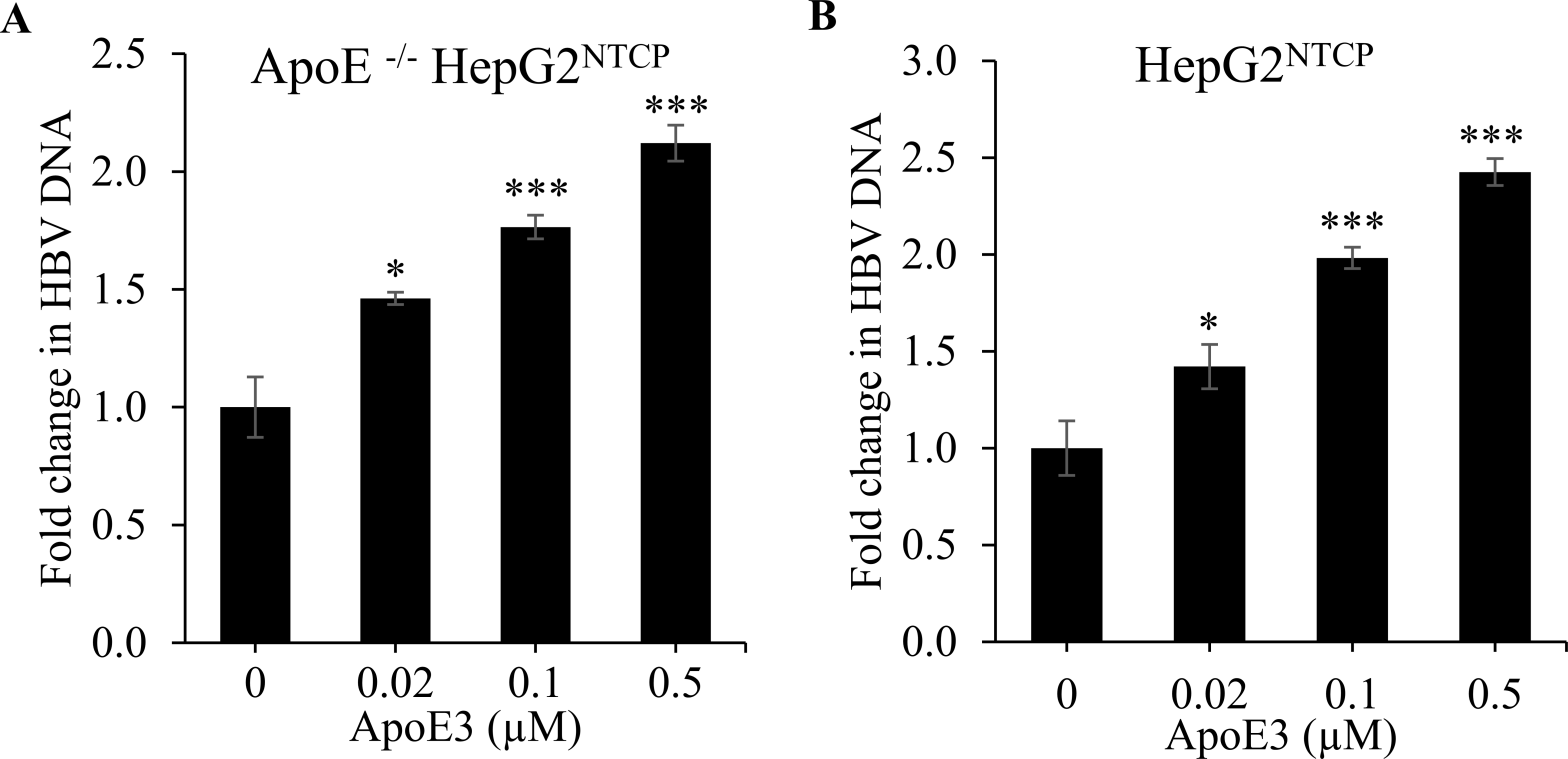
Determination of extracellular apoE3-mediated HBV cell attachment. HBV was mixed with varying concentrations (0, 0.02, 0.1, and 0.5 µM) of apoE3-containing cell culture supernatants at RT for 30 min. The mixtures of HBV and apoE3 were then incubated with apoE^-/-^ HepG2^NTCP^ (**A**) or parental HepG2^NTCP^ cells (**B**) on ice for 6 h. The unbound HBV was removed by extensive washing with 1× PBS. HBV-bound cells were subjected to DNA extraction. The levels of HBV DNA were quantified by a qPCR method. The levels of HBV DNA relative to HBV in the absence of apoE3 were calculated and presented as fold of change. Data represent the mean ± SD from triplicate experiments. *P < 0.05, ***P < 0.001.

Our previous studies with HBV and HCV demonstrated that apoE mediates HBV and HCV cell attachment (24). To further define the specific step of apoE-mediated promotion of HBV infection, an HBV cell attachment assay was carried out by incubating HBV with either apoE-deficient (Fig. 7A) or parental HepG2^NTCP^ cells (Fig. 7B) in the presence of increasing concentrations of extracellular apoE3 in the cell culture supernatants on ice for 6 h. The unbound HBV was removed by thoroughly washing cells with PBS. Total DNA was extracted from HBV-bound cells, and HBV DNA was quantified by a real-time qPCR method using HBV-specific primers and probe. The data show that extracellular apoE3 similarly promoted HBV attachment to apoE-deficient and parental HepG2^NTCP^ cells in a dose-dependent manner, consistent with our previous findings that apoE enriched on the viral envelopes facilitates HBV and HCV cell attachment.

## DISCUSSION

Human apoE is a plasma exchangeable apolipoprotein important for lipid and cholesterol transport and metabolism, cell growth and differentiation, regulation of host immunity, host repair response to tissue injury, and Alzheimer’s disease development (20, 35). It is also involved in the life cycle and/or pathogenesis of many different viruses as either a proviral or restriction factor. In many cases, apoE is identified as a proviral host factor promoting viral infection, replication, and/or pathogenesis, including but not limited to HSV-1 (36), MHV68 (22), HBV (24), and HCV (25). In contrast, apoE was found to restrict HIV and influenza virus infection and/or replication through targeting HIV envelope protein for degradation or mediating cell resistance to influenza virus infection (23, 37). ApoE may also play an important role in viral pathogenesis via modulation of host immune response and/or regulation of lipid and cholesterol homeostasis, as well as in the maintenance of target cell membrane integrity (37–39). In the case of HBV and HCV, we have previously demonstrated that apoE has dual functions in viral infection and virion production through distinct mechanisms (24, 25). ApoE recruitment to HBV and HCV envelopes, as well as apoE expression on the target hepatocytes, facilitate viral infection through apoE-mediated cell attachment (24, 30). The lipid-binding domain of apoE interacts with viral envelopes and/or structural proteins to promote virion assembly, envelopment, maturation, and/or release, resulting in an increase of virus production, as suggested by the studies with HSV-1 and HCV (24, 30, 36, 40).

In the present study, we have uncovered a previously unrecognized mechanism of action of apoE in the promotion of HBV infection through the rapid transfer of extracellular apoE to the HBV envelope. Extracellular apoE efficiently transferred to HBV envelope upon incubation with an apoE-deficient HBV, as determined by reciprocal pulldown of HBV and apoE using HBsAg- and apoE-specific antibodies (Fig. 1), as well as shown by apoE transferred to HBV upon purification (Fig. 4A). The use of apoE-deficient HBV allows the tracking and detection of extracellular apoE transferred to HBV envelope as well as the determination of defective HBV infectivity restored by extracellular apoE. The transfer of extracellular apoE to HBV is in line with the intrinsic property of apoE as an exchangeable apolipoprotein that binds to the lipid surface of lipoproteins and readily transfers between lipoprotein particles (HDL, LDL, VLDL, and chylomicrons) (33, 41). The envelope of HBV resembles the lipid surface of lipoproteins. It is also possible that HBV is associated with lipoproteins or assembled as a lipoviral particle (LVP), as previously shown for HCV (42–44). Future structural and/or electron microscopic studies are warranted to further determine how apoE interacts with HBV envelope *in vitro* and *in vivo*. More importantly, transfer of extracellular apoE to the HBV envelope fully restored the defective infectivity of apoE-deficient HBV. ApoE3 lipoproteins produced from apoE3-overexpressing HepG2 cells as well as from apoE3-KI mice significantly enhanced HBV infection in dose-dependent manner, as determined by increased numbers and intensity of HBcAg-positive cells and higher levels of L-HBsAg expression in HBV-infected cells, as well as elevated levels of HBeAg and HBV DNA in the cell culture supernatants (Fig. 2 and 3). By contrast, cell culture supernatants from apoE-knockout HepG2 did not significantly affect HBV infection, suggesting that the enhanced HBV infectivity was ascribed to extracellular apoE transferred to HBV. These findings were further validated by the results derived from the studies with purified HBV with or without apoE enrichment. Purified HBV with apoE3 transfer had 3-fold to 4-fold higher infectivity compared to purified apoE-deficient HBV (Fig. 4). More importantly, a purified recombinant apoE3 that was lipidated with POPC significantly promoted HBV infectivity when mixed with apoE-null HBV (Fig. 5). Additionally, the infectivity of regular HBV was also enhanced by extracellular apoE3 lipoprotein (Fig. 6). However, apoE3 did not significantly affect HBV replication in cell culture, as also demonstrated by our previous studies (24). Collectively, these findings suggest that extracellular apoE3 lipoproteins may play a critical role in HBV infection and pathogenesis *in vivo*. For instance, the levels of apoE lipoproteins in humans may determine the outcomes of HBV infection or viral persistence in chronic hepatitis B patients.

The question arose as to how extracellular apoE3 promotes HBV infection. The findings from this study clearly show that HBV cell attachment was significantly enhanced by the transfer of extracellular apoE3 to the HBV (Fig. 7). This mechanism is also supported by the findings obtained from the studies with HSV-1 (23) and HCV (30, 32). ApoE on the virus envelopes mediates HBV binding to the cell surface of hepatocytes through apoE-binding receptors such as HSPGs and LDLR family receptors. It was previously shown that heparin or treatment of cells with heparinase decreased HBV infection (13, 15). HSPG core proteins, such as GPC5 and SDC2, were also identified as HBV attachment receptors (16, 18). Besides HSPGs, we found that LDLR is also important for HBV infection by mediating HBV cell attachment (14). Additionally, apoE may also facilitate virus entry, as suggested by the studies with HSV-1 (23). It is conceivable that apoE binding to its receptors on the cell surface would promote HBV endocytosis (45). This may explain the discrepancy between apoE3-mediated HBV infection and cell attachment. Extracellular apoE3 enhanced HBV infection by up to 4-fold to 6-fold but HBV cell attachment by only 2-fold to 2.5-fold (Fig. 2 and 7). HBV-associated apoE may have additional functions in the HBV life cycle and/or pathogenesis in hepatitis B patients by promoting HBV spread, virion stability, and persistence in circulation. So far, existing cell culture models of HBV infection do not support HBV propagation and consequently are unable to examine HBV cell-to-cell spread in cell culture. It will be intriguing to determine if the levels of circulating apolipoproteins in the plasma (hypercholesterolemia) of chronic hepatitis B patients correlate with HBV viremia and/or liver disease severity, which warrants further clinical investigations in the future.

## MATERIALS AND METHODS

### Cell culture

NTCP-expressing HepG2 (HepG2^NTCP^), HBV-producing HepAD38, apoE-knockout HepAD38, and apoE-knockout HepG2^NTCP^ cells were described previously (24, 46). They were cultured in DME/F12 medium (Hyclone) supplemented with 10% fetal bovine serum (Gemini), 100 U/mL penicillin, 100 U/mL streptomycin (Corning), 1× MEM non-essential amino acids (Corning), and 1× sodium pyruvate (Corning). HepAD38 cells were maintained in the presence of 250 μg/mL of G418 and 1 μg/mL of doxycycline (Fisher Scientific). ApoE-knockout (apoE^-/-^) HepG2^NTCP^ cell line overexpressing a human apoE3 was maintained in the presence of blasticidin (5 µg/mL) and G418 (500 µg/mL). All culture flasks and plates were pre-coated with 50 μg/mL rat tail collagen type I (Corning, 354236).

### Antibodies and reagents

HBcAg-specific monoclonal antibody C1–5 was purchased from Santa Cruz (sc-23945). HBV preS1-specific monoclonal antibody (7H11) was kindly provided by Shuping Tong (Brown University). HRP-conjugated goat anti-mouse antibody was obtained from Invitrogen. ApoE-specific monoclonal antibody (WUE4) and MAb23 were made in the lab, as described previously (25). Human GAPDH (6C5) monoclonal antibody was purchased from Sigma-Aldrich. Rapid Gold BCA Protein Assay reagents were from Thermo Scientific (A53225). Alexa Fluor 594-conjugated goat anti-mouse IgG was from Invitrogen. HBeAg enzyme immunoassay (EIA) kits were purchased from International Immunodiagnostics (USA). DNA isolation kits (DNeasy Blood and Tissue, Cat No. 69506) were purchased from Qiagen. Clarity Max Western blotting ECL substrate was purchased from Bio-Rad. DMRIE-C reagent was obtained from Invitrogen. Dimethyl sulfoxide (DMSO), polyethylene glycol (PEG) 8,000, hydrocortisone, and co-IP kit (IP50) were all obtained from Sigma-Aldrich. iTaq qPCR master mix was purchased from Bio-Rad. A purified recombinant human apoE3 was purchased from PeproTech. The 1-Palmitoyl-2-oleoyl-sn-glycero-3-phosphocholine (POPC) was purchased from Avanti Polar Lipids, Inc.

### HBV production and concentration

HBV-producing HepAD38 cell lines with and without apoE-knockout were cultured in DME/F12 medium supplemented with 3% tetracycline-negative fetal bovine serum (Gemini) and 1% DMSO in HyperFlasks (Corning), as described previously (14). Culture supernatants were harvested every 3 days and were passed through 0.45 μm filtration units (Corning). HBV was concentrated by centrifugation with Amicon Ultra Centrifugal Filters (Millipore Sigma, UFC910096). HBV DNA genome copy numbers were quantified by a real-time PCR method using primers and probe as described previously (14, 24).

### Co-immunoprecipitation of HBV and ApoE

Co-immunoprecipitation of apoE and HBV virions was performed using a co-IP kit according to the manufacturer’s protocol (Sigma-Aldrich). Briefly, HBV was incubated with concentrated cell culture supernatants of an apoE3-overexpressing HepG2 cell line at room temperature (RT) for 6 h. The HBV/apoE3 mixtures were subjected to co-IP using an apoE monoclonal antibody (MAb23), hepatitis B immune globulin (HBIG), normal mouse, or human IgG at 4°C overnight. Protein G agarose beads were then added for an additional 2-h incubation at 4°C. Upon washing of agarose beads, immunoprecipitated complexes were eluted by boiling in 1× Laemmli sample buffer at 95°C for 5 min, followed by electrophoresis in 4%–20% precast protein gels (Bio-Rad). HBcAg and apoE3 were determined by western blotting using HBcAg- and apoE-specific antibodies.

### HBV purification

ApoE-deficient HBV and its mixture with apoE3-KI mouse serum were purified by cesium chloride (CsCl) gradient ultracentrifugation as previously described (24). The CsCl gradient was fractionated from the bottom to the top. A total of 16 fractions were collected and numbered from top (F1) to bottom (F16). The levels of HBcAg and apoE in each fraction were determined by western blot analysis, whereas HBV DNA in each fraction was quantified by qPCR upon DNA extraction with a QIAGEN DNA isolation kit. Fractions containing most HBV virions were used for infection of HepG2^NTCP^ cells at a multiplicity of infection (MOI) of 100 HBV genome equivalents (100 GEq). After 4 days p.i., HepG2^NTCP^ cell lysates were used for the detection of L-HBsAg by western blot analysis. The levels of HBeAg and HBV DNA in the cell culture supernatants were quantified by EIA and qPCR, respectively.

### Preparation of extracellular apoE3 lipoproteins

Human apoE3-knockin (KI) mice B6.Cg-Apoe^em2(APOE*)Adiuj^/J (Jax strain 029018) were obtained from Jackson Laboratory. Mouse blood was collected upon euthanasia with isoflurane. Mouse serum was separated by centrifugation at 2,500 × *g* for 30 min. Mouse serum samples from different apoE3-KI animals were combined, aliquoted, and stored at an ultralow temperature (−80°C) freezer. To prepare apoE3 lipoprotein in cell culture, a human apoE3-expressing plasmid DNA vector pcDNA3.1/hApoE3 (a gift of Theodore Mazzone, University of Illinois at Chicago) was transfected into an apoE-knockout HepG2 cell line (24, 29). ApoE3-overexpressing HepG2 cell clones were selected with G418 (500 μg/mL) and detection of apoE3 by WB using apoE monoclonal antibody WuE4. Cell culture supernatants of HepG2 with the highest apoE3 expression were concentrated by passing through 10 kDa Amicon Ultra centrifugal filters (Millipore). ApoE3 in mouse sera and cell culture supernatants were quantified by the EIA method using a purified recombinant apoE3 as a standard (PeproTech). To reconstitute apoE3 lipoprotein, a purified recombinant ApoE3 was mixed with POPC at a molar ratio of 1:50 in PBS, followed by sonication and incubation prior to its use (34).

### HBV infection

HepG2^NTCP^ cells were seeded at a cell density of 2 × 10⁵ per well in 24-well cell culture plates (Corning) and 1 × 10⁵ per chamber of 8-well µ-slides (ibidi) for IFA. Cells were then infected with HBV at an MOI of 600 genome equivalents for ApoE-deficient HBV or 200 for regular HBV produced from HepAD38 in the presence of 4% PEG, as described previously (14, 18, 24, 29). After 12 h of incubation, the unbound HBV was removed, and the HBV-infected cells were washed with PBS three times. HBV-infected HepG2^NTCP^ cells were incubated with DME/F12 medium containing 3% FBS, 1% DMSO, and 5 μg/mL hydrocortisone for 4 days. HBcAg and L-HBsAg in HBV-infected cells were determined by IFA and western blotting, respectively. HBeAg and HBV DNA in cell culture supernatants were quantified by EIA and qPCR, respectively.

### Immunofluorescence assay (IFA)

HepG2^NTCP^ cells in µ-slides were fixed with 4% paraformaldehyde for 20 min, permeabilized with 0.1% Triton X-100 for 8 min, and blocked with 3% BSA-PBST overnight at 4°C. HBcAg in HBV-infected cells was stained with a HBcAg-specific monoclonal antibody C1-5 (1:100 dilution) at 4°C overnight, followed by Alexa Fluor 594-conjugated goat anti-mouse IgG (1:2,000) for 1 h at room temperature. Nuclei were stained with DAPI (1 μg/mL) for 10 min. Images were captured using a fluorescence microscope and processed ImageJ.

### Western blot analysis

HepG2^NTCP^ cells were lysed in a RIPA buffer. Protein concentrations in cell lysates were determined using BCA reagent. A total of 50 μg protein each was loaded onto 12% SDS-PAGE gels. Upon electrophoresis, proteins were transferred onto polyvinylidene difluoride (PVDF) membranes using a semi-dry transfer system (Bio-Rad). The membranes were blocked with 5% nonfat milk and then incubated with a preS1-specific monoclonal antibody (7H11) at 4°C overnight. GAPDH was used as a housekeeping gene control. L-HBsAg and GAPDH were detected using an HRP-conjugated goat anti-mouse IgG and ECL substrate (Bio-Rad). Protein bands were visualized with the ChemiDoc MP Imaging System (Bio-Rad).

### Quantification of HBeAg by an enzyme immunoassay (EIA)

The levels of HBeAg secreted to cell culture supernatants were determined by EIA kits (International Immunodiagnostics). Absorbance was measured at a wavelength of 450 nm using a BioTek plate reader. The levels of HBeAg were calculated and converted as international units per milliliter (IU/mL) based on the titration curve using a recombinant HBeAg as a standard.

### HBV DNA isolation and quantitative PCR (qPCR)

HBV genomic DNA was isolated from cell culture supernatants using DNeasy Blood and Tissue kits (Qiagen). HBV DNA was quantified by qPCR using HBV-specific primers: 5′-GAGTGTGGATTCGCACTCC-3′ (forward) and 5′-GAGGCGAGGGAGTTCTTCT-3′ (reverse) and probe (5′-FAM-CCGTGTGCACTTCGCTTCACCTCTGC-TAMRA-3′) (18). qPCR was conducted using iTaq master mix and Bio-Rad instrument at 95°C for 3 min and 40 cycles at 95°C for 15 s and 60°C for 1 min.

### HBV cell attachment assay

ApoE-null HBV was mixed with increasing concentrations of extracellular apoE3 present in cell culture supernatants at room temperature for 30 min prior to incubation with parental or apoE-deficient HepG2^NTCP^ cells. After a 6-h incubation on ice, the unbound HBV was removed, followed by extensive washing with 1× PBS. Cell-bound HBV was subjected to DNA extraction with the QIAGEN DNeasy isolation kit. The levels of HBV DNA were quantified by qPCR.

### Statistical analysis

Statistical analyses were performed using GraphPad Prism 8 software. Data were expressed as means ± standard deviations (SD) calculated from triplicates. The differences between groups were compared using a paired two-tailed *t*-test. A *P*-value of less than 0.05 (*P* < 0.05) was considered statistically significant. *P < 0.05, **P < 0.01, ***P < 0.001.

## Data Availability

Original data derived from the present study and experimental methods are fully available on request.
